# The connected workplace: Characteristics and social consequences of work surveillance in the age of datification, sensorization, and artificial intelligence

**DOI:** 10.1177/02683962231202535

**Published:** 2023-09-22

**Authors:** Tobias Mettler

**Affiliations:** 27213 University of Lausanne, Switzerland

**Keywords:** Artificial intelligence, algorithmic management, datification, control theory, future of work, physiolytics, sensors, surveillance capitalism

## Abstract

Because of COVID-19 lockdowns, managers and administrators have begun to look for new ways to monitor and control their stranded-at-home workforce. Yet long before the pandemic already, advancements in datification, sensorization, and artificial intelligence have given rise to what we call *connected workplace surveillance*. At the heart of this new mode of employee monitoring and control is the extension of the scope of data collection beyond what is necessary and reasonable for performance appraisals or managerial oversight. This includes treating an employee’s body as a data source, disrespecting the boundaries between business and private life, or using gathered surveillance information for subtle persuasion, manipulation, and coercion. This article provides a new perspective on control theory, examining the characteristics of connected surveillance and comparing it to visual or computerized surveillance. Taking an employee-centric position, it also proposes a research agenda for critical, behavioral, and design-oriented scholars who wish to explore the identified issues.

## Introduction

Owing to the COVID-19 pandemic, millions of people suddenly stopped going to their workplaces and started doing their jobs from home. While this crisis has accelerated the adoption of remote work (Baig et al., 2020) and has forced employees to adapt their working styles (Waizenegger et al., 2020), it had little impact on the popularity of and continued insistence on Taylorist management styles (Wang et al., 2020). Worse yet, being unable to directly monitor and surveil their employees, the lockdowns of businesses around the world have highlighted one of the worst fears of managers and administrators: loss of control.

Therefore, it is no coincidence that we are seeing a surge in new work surveillance technologies (Putzier and Cutter, 2022). Promising to make employees happier, more loyal, more productive, collaborative, and innovative (Franklin, 2021), tech companies have developed highly sophisticated systems, going beyond simply recording employees’ digital traces, such as keyboard strokes, mouse movements, or website and file histories (Harari, 2020; Satariano, 2020). Intending to reduce an employee to a comprehensive score (Leonardi and Contractor, 2018), or to provide instant feedback (Rivera et al., 2021), these—what we call—*connected workplace surveillance* solutions scrutinize and integrate unprecedented amounts of work-related and non-work-related personal data. In a time where it seems socially accepted and politically desired for companies to act like *private governments* (Anderson, 2017), self-regulating and contained only by weak enforcement of laws (De Stefano, 2020), one would expect little resistance to new work surveillance types.

Companies that are adopting or designing connected workplace surveillance solutions nonetheless experience value conflicts and discursive struggles similar to how, 40 years ago, computerized performance monitoring systems (CPMS) (Grant and Higgins, 1991; Irving et al., 1986) sparked a first heated debate on workers’ rights to privacy and work dignity (Kling, 1996; Mason, 1986). Microsoft, after facing severe public criticism over its Productivity Score—a tool supposed to help organizations measure and manage the use of its Microsoft 365 applications suite—had to back down and remove all users’ names and all measures that quantify individuals’ user behaviors (Spataro, 2020). Amazon was confronted with the largest, most viable unionization effort of its U.S. warehouse workforce, among other things, owing to its introducing a new employee tracking technology (Corkery and Weise, 2021). Why this unexpected outcry? The fact that our work is being monitored, recorded, tracked, and controlled is not a recent phenomenon, as it goes back to the emergence of capitalism, with the shift from subsistence labor on farms to hourly and salaried work in factories and offices (Beniger, 1989). Work surveillance has evolved gradually with changing technologies and workspace designs: time clocks and punch cards were followed by time reporting and transaction monitoring with spreadsheets and then sensor networks, wearable devices, body implants, and artificial intelligence (AI). What has changed to provoke such a reaction?

This article seeks to outline how work surveillance has changed owing to the increased use of datification, sensorization, and AI as well as to propose a research agenda and three testable propositions that will be useful in uncovering the possible social consequences of the introduction of these technologies. A central tenet is that the connected workplace crosses a line that previous work surveillance types did not. Being an ensemble artifact (Sein et al., 2011) composed of distinct hardware and software components, next-generation work surveillance systems are well equipped to extend the scope of their surveillance beyond what workers do in front of their computer monitors. Datification, sensorization, and AI not only enable more varied, pervasive, and widespread monitoring practices but also make it palpably easier to decipher intimate preferences, everyday routines, subjective well-being, or sentiments toward their employer to the extent of predicting resignations (Fang et al., 2018) or job burnout (Dai and Zhu, 2021). On the one hand, these tools can benefit workers, helping to prevent serious accidents (Sarkar et al., 2019) and helping to protect them from life-threatening hazards (Asadzadeh et al., 2020) or damages owing to unhealthy work habits (Ailneni et al., 2019). On the other hand—and the focus of this article—the connected workplace poses risks to workers’ fundamental rights and dignity. Since the boundaries of what constitutes a workplace are becoming increasingly porous, these tools not only track employees’ (online and offline) behaviors, their health status, or the frequency of their rest breaks during working hours but also when employees are supposedly off-the-clock or when they are working remotely from home (De Vaujany et al., 2021). Given that ordinary employees have received little scholarly attention (Giermindl et al., 2022), we deliberately focus on a set of broad research directions from an employee-centric perspective. We thus follow in the footsteps of research, such as the Scandinavian “trade-unionist approach” to systems design (Iivari and Lyytinen, 1998) which, rather than comprehending organizations as value-neutral and harmonious assemblages of people, assume a subliminal conflict between the interests of labor and capital (Bødker et al., 1987; Hedberg, 1980; Sandberg, 1985).

This article makes two primary theoretical contributions. First, we offer a conceptualization and a deeper understanding of the characteristics of the connected workplace. As we show, contemporary work surveillance is no longer limited to monitoring, recording, or tracking but in most instances also incorporates obvious or hidden, benevolent or exploitative, reinforcing or reprogramming behavioral strategies that help trigger modifications of employees’ attitudes, perceptions, motivations, and actions (Díaz Andrade and Techatassanasoontorn, 2021)—for good or bad. We argue that the growing controversy about the connected workplace is fueled by the fact that these new work surveillance types go beyond what is reasonable, often collecting more personal data that is veritably necessary (or legal) for performance appraisals and managerial oversight (Ball, 2021), to the extent to which the connected workplace not only becomes a nuisance but also negatively affects employees’ levels of self-determination, autonomy, choice, trust and—eventually—an organization’s overall productivity.

Second, we open a new debate about the essence of control theory. While most of the IS literature has capitalized on the idea that control is most effective when enacted through social contracts, agreements, and arrangements (Huang Chua and Myers, 2018; Kirsch et al., 2002), we posit that businesses that implement connected workplace surveillance often tend to adopt an *organization-as-a-machine* worldview that treats control less as a form of a social enactment, but as a cybernetic cycle or well-defined set of mappings between inputs and outputs as well as causes and effects. Based on the cybernetic view of control, as defined by Lord and Hanges (1987), we demonstrate how control in a connected workplace differs from previous work surveillance modes.

The remainder of the article is structured as follows. In the next section, we describe the historical evolution of work surveillance and how new technologies trigger a so-called *control crisis*. Assuming that datification, sensorization, and AI will trigger fundamental changes to a similar extent as mechanization and computerization did, we then outline the key properties of contemporary types of connected surveillance. Subsequently, we focus on understanding the conception of control in a connected workplace. This is followed by a discussion on the potential social consequences that result from the implementation of this understanding of control. We conclude with a proposed research agenda for the IS community, to expand our knowledge of and develop responses to the negative social consequences identified in our analysis.

## History of work surveillance

Most historical accounts of work surveillance go back to the emergence of capitalism (Zuboff, 2015). Different from previous modes of production (e.g., serfdom and patrimonialism), a key distinguishing characteristic of capitalism is its reliance on markets and competition, which ultimately led to human labor becoming a commodity for sale (Manokha, 2020). As Marx (1976) noted, “*The purchaser of labour-power consumes… by setting the seller of it to work*” to best exploit their investments by limiting any underperformance or waste. Thus, and deliberately interfering with an employee’s privacy and integrity, the employer needs to set up some type of monitoring and performance appraisal to gain an overview over the allocation of resources and profit maximization. Following Taylor (2003), this is best performed with scientific accuracy and rigor.

### The first control crisis

Clocking in, counting, and weighing output and payment by means of piece-rates became particularly important when new factory production methods (e.g., assembly lines and conveyor belts) superseded the slower, human pace of labor (Ball, 2010). According to Beniger (1989), this led to *the first control crisis* as employers suddenly had to process information at industrial speed to keep up with performance monitoring. Since in the Taylorist worldview an unobserved employee is an inefficient one, a non-technical measure, which persists until today, was to divide the workforce into laborers and overseers with the intention that the latter prevent the former from slowing or sabotaging the modes of production (Saval, 2014). A technical measure was to use portable and precise mechanical clocks with which “*the full abstraction of work time into commodified hours*” could be captured (Snyder, 2016).

Early work surveillance modes primarily centered around *visual surveillance* practices, limited to the overseers’ oversight of employees’ behaviors and outcomes in the premises where work is performed (Zureik, 2003). A prominent literary example of the idea that control can be exerted through *gazing* has certainly been Orwell’s (2000) novel *1984*, where Big Brother’s physical absence yet psychological ubiquity creates a sense of hopelessness and futility to commit any misbehavior. The power of the gaze has also been intensively studied in research (Ball and Wilson, 2000; De Moya and Pallud, 2020; Willcocks, 2004). The most prominent example has been Foucault’s (1973) study on how the architectural, *panoptic* design of institutions such as asylums and hospitals could be arranged so that the overseers’ power to invigilate and control the behaviors of the watched (e.g., employees, prisoners, and patients) is optimal, while their visibility is minimal.

### The second control crisis

**Figure 1.**
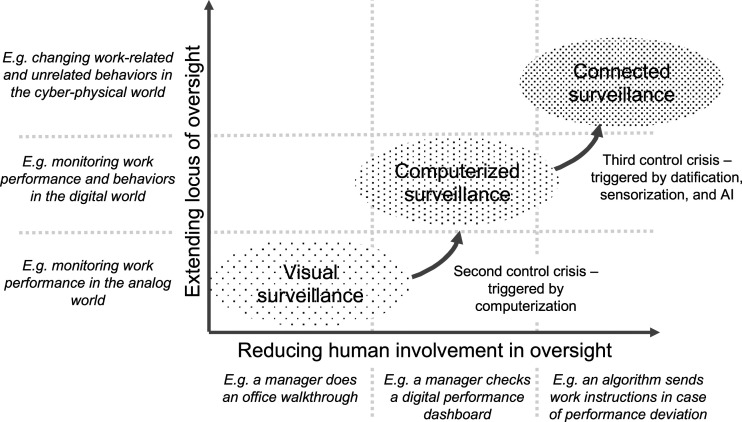
Historical development of work surveillance.

With work shifting from factory halls to office cubicles, and employees operating in front of computers instead of workbenches, it became increasingly difficult for employers to determine performance only through gazing. Alongside the introduction of enterprise resource planning systems, this prompted many companies in the early 1980s to implement CPMS, which in turn stimulated a heated debate on the ethical limits of *computerized surveillance* at the workplace (Irving et al., 1986; Mason, 1986; Zuboff, 1988). After the National Association of Working Women reported that, in 1984, an estimated 20% of clerical employees are being monitored by computers (Grant and Higgins, 1989), theU.S. Government Office of Technology Assessment (1987) published the multidisciplinary report *The Electronic Supervisor: New Technologies, New Tensions*, which rated the proportion of workers under computerized surveillance to be even higher—approximately 25% to 35%. It concluded that, while such systems may be beneficial for employers for measuring job efficiency and overall productivity, they also come at the expense of the quality of an employee’s work life. Although the word *technostress* (Ragu-Nathan et al., 2008) was not explicitly used, this report provided initial evidence of the potentially harmful consequences of computerized surveillance owing to increased pressure, particularly among under-trained employees, with low job security, or whose wages depend on measurement scores. Some years later, Grant and Higgins’s (1991) study demonstrated that computerized surveillance does not necessarily result in productivity increases. Hawk (1994) reported somewhat puzzling findings, showing that CPMS do not inevitably lead to more stressful workplaces but rather negatively affect the perceived fairness of appraisal. A common assumption in the mentioned studies, and subsequently conducted ones, has been that computerized surveillance only takes place while working at the company’s premises, with company property, or during working hours (Nord et al., 2006). As we will argue, this assumption is no longer valid because today’s connected workplaces are not limited to the spatial and temporal boundaries of traditional work. It also opens new and sometimes conflicting questions about the future of (human) managers and the very nature of managerial oversight (see Figure 1). We will now look closely at the connected workplace’s characteristics and the changes that *the third control crisis* will bring about.

## Characteristics of the connected workplace

### The third control crisis

The third control crisis is latent and is gradually becoming experienced by millions of people who, owing to the COVID-19 pandemic, are or were forced to work from home. As if in a natural experiment, employers are trying new ways to monitor their stranded-at-home workforce (Heaven, 2020; Putzier and Cutter, 2022). Different to previous computerized surveillance types, which mainly measured work-related online activities (e.g., keystrokes, computer time usage, and committed transactions), the new wave of surveillance tools also accumulates non-work-related, personal, and sensitive data (e.g., what an employee believes, likes, and how well/fit/healthy they are), similar to how tech companies have scrutinized consumers’ online behaviors (Clarke, 2019; Zuboff, 2015). Companies such as Amazon (acquiring One Medical), Google/Alphabet (acquiring Fitbit, Nest, and Senosis), or Facebook (acquiring CTRL-Labs and FacioMetrics) have long been investing in new technologies that extend their capabilities to capture user behaviors and characteristics beyond what happens on a computer screen. Similarly, companies that specialize in work surveillance have shifted their attention from mass or group surveillance to much more personalized behavioral surveillance types (Chen and Ross, 2007). For instance, Isaak, a UK-based AI solution, seeks to provide employers with minute-to-minute information about their workforce by harvesting data on who e-mails whom and when, who accesses and edits files, and who meets whom and when. According to Status Today (2022), the company behind Isaak, its solution should enable employers to identify employees who are “*change-makers*” in the organization. The Boston-based company Humanyze integrates information from multiple collaboration tools and smart office sensors (e.g., sociometric badges that employees must wear during work time) with the promise to “*rapidly validate the impacts of business strategies to drive the desired outcomes*” (Humanyze, 2022). Enaible. io (also located in Boston) has designed an algorithm that quantifies employees’ productivity through a “*multi-dimensional calculation of capacity utilization, consistency and quality impact*” (Enaible.io, 2022). The abovementioned examples have several common characteristics, which we will now describe.

### Every employee’s body as a data source

Intending to take full control of an employee as a resource and to predict variations in productivity, employers have extended the scope of data collection beyond monitoring only work activities (Kamal, 2020). According to the European General Data Protection Regulation (GDPR), organizations are allowed to process personal data without requiring any explicit consent from their employees “*for the purposes of preventive or occupational medicine, for the assessment of the working capacity of the employee, medical diagnosis, the provision of health or social care or treatment or the management of health or social care systems and services [….]*” (Art. 9.2h GDPR). In this sense, under the GDPR, an employer can use an employee’s health data if it can prove that such processing is necessary for improving safety and well-being in the workplace (Amankwah and Stroobants, 2022; Forcier et al., 2019). Thus, an employee’s body becomes both a data source that needs to be monitored, assessed, analyzed, and categorized (Moore, 2018; Van der Ploeg, 2012) as well as a risk factor that needs to be contained, managed, and optimized (Berry et al., 2010; Mettler and Wulf, 2019). In practice, this trend manifests in two ways.

First, following the success of quantified-self practices in the consumer market (Agarwal and Dhar, 2014), companies have begun to invest heavily in occupational health and wellness programs (Gorm and Shklovski, 2016; Vyas et al., 2015; Yassaee and Mettler, 2017), which rely on a variety of devices (e.g., badges, patches, rings, wristbands, and smartwatches) that link the measurement of body functions (e.g., pulse, sweat, and respiration) and behaviors (e.g., physical activity and calorie intake) to algorithmic decision-making (see Table 1). Assuming that health data collection will not only benefit the employer's goal to predict future sickness absences but also generate immediate value to employees, such as for managing their work-related stress or improving their physical and psychological well-being, these devices’ transformation potential and predictive power remain limited (Stein et al., 2020). Nonetheless, about 27.5 million fitness devices were sold in 2020, compared to only 166,000 in 2013 (Olson, 2022). The pandemic has accelerated this upward trend owing to regulatory needs for complementary measures (e.g., digital contact tracing) so that employees can safely return to work (Cox, 2020; Kudyba, 2020).

**Table 1.** Studies that exemplify the conception of the quantified workplace and quantified employees (Mettler and Stepanovic, 2023).

Issue	Data collected	Exemplary studies
Physical inactivity, sedentary behavior, and movement habits	Step counts, distances, body movement gathered using activity trackers, thermal sensors, etc.	Glance et al. (2016); Gomez-Carmona and Casado-Mansilla (2017); Gorm and Shklovski (2016); Nair et al. (2019); Synnott et al. (2016)
Physical pain and bad posture	Neck movement, lower back movement, head movement, seat surface, backrest monitored by smart cloth, smart furniture, etc.	Lo Presti et al. (2020); Roossien et al. (2017); Zaltieri et al. (2020)
Psychological well-being, absenteeism, and burnout	Heart rate, skin temperature, skin blood perfusion, blood oxygenation, respiration rate, heart rate variability, blood pulse wave, speech and voice tones, body posture, hand gestures, nutritional information gathered by wearable biosensors, smartphones, sensor networks, etc.	Bhatia and Sood (2019); Fugini et al. (2020); Stepanovic et al. (2019); Zenonos et al. (2016)
Environmental health hazards such as poor air quality, excessive heat or humidity, and fire risks	Ambient light intensity, radiant or air temperature, relative humidity, carbon dioxide level, desk occupancy, desk cleanliness, background noise, number of phone calls monitored by smartphones, sensor networks, etc.	Benhamida et al. (2019); Nižetić et al. (2020); Rabbani and Keshav (2016)

Second, fueled by the popularity of electronic fingerprints, hand geometry, face recognition, and other identity access management approaches applied in consumer electronics, more and more companies have begun to systematically record certain biometric information about their employees (Ball, 2010). This has progressed to the point where these data are no longer used only for identity and access control but also as modern-day punch clocks that register an employee’s attendance as well as their physical and digital movements (Brooks, 2020), or for operating company devices and equipment made possible by rice grain-sized radio frequency identification skin implants developed by the Swedish company Epicenter (Rothschild, 2020). Signing a *biometric consent form* has become a requirement for Amazon drivers, so that the surveillance system in its trucks can access drivers’ location, movement, and biometric data (Gurley, 2022).

Treating an employee’s body as a data source and extending the scope of data collection beyond purely work-related activities have several implications. On the one hand, it requires employers to increase their privacy and security protocols (Classen et al., 2018). On the other hand, it drastically shifts work surveillance’s focus from fairly impersonal mass or group-level monitoring (e.g., video cameras in office buildings) to fairly personal and sensitive behavioral tracking and prediction (e.g., individual health scores and predicted burnout rate), transforming the ways employers interact with employees as the workplace increasingly begins to resemble a professional sports club (Day et al., 2012).

### Shifting the locus of work surveillance

It is not only surveillance technologies and management practices that have changed since the 1980s, the very nature of work has also changed. Offering jobseekers opportunities in remote geographic areas or to absorb short-term economic downturns in the offline economy (Huang et al., 2020), on-demand or the so-called gig platforms such as Amazon Mechanical Turk, Deliveroo, Handy, and Uber have made work more flexible, mobile, and informal, but also more short-term and uncertain (Benson et al., 2019). Notwithstanding the fiscal, labor law, and social security issues caused by the gig economy in recent years (Graham et al., 2020), this increasing flexibilization of work has blurred the boundaries between business and private life and has moved work from company property (e.g., the ubiquitous work computer) to the tools and hardware that gig workers have at their disposal (e.g., personal smartphones and tablets). Since platform owners do not know their workers, they rely on both customers overseeing and appraising a gig worker’s behaviors as well as surveillance technologies that continually trace and log activities, movements, or communication. Thus, work surveillance is not limited to either the physical sphere (the first control crisis) or the digital sphere (the second control crisis) but combines and integrates different sources of information, which help to get an overview over what happens both online and offline (see Table 2).

**Table 2.** Studies that exemplify a shift in the locus of work surveillance.

Issue	Data collected	Exemplary studies
Tracking of (offline) workers (e.g., taxi drivers, cleaning staff, and assembly line workers)	Geolocation, time spent at a location, movement, speed, dangerous or improper behaviors (e.g., texting while driving), assignment completion time, etc.	Bednar and Welch (2020); Rosenblat and Stark (2016); Wiener et al. (2021); Zhang et al. (2020)
Tracking of (online) workers after working hours or outside the company’s premises	Geolocation, activity pattern at the home office, text messages, personal social media posts, email, other written communication, etc.	Faraj et al. (2021); Lee (2011); Stanko and Beckman (2014); Wang et al. (2022)

**Figure 2.**
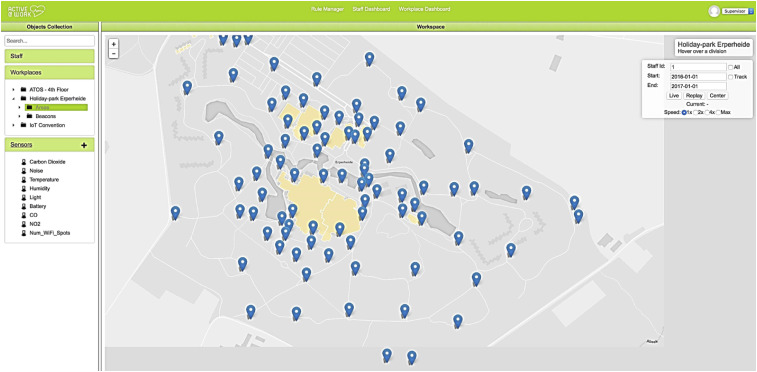
A work surveillance application that tracks the physical locations of cleaning staff in an amusement park (picture obtained from a project that the author participated in).

Yet such practices are not limited only to the gig economy. Apps with a GPS function that allow for the tracking of an employee’s whereabouts—such as Xora or StreetSmartWorkforce—are used by all sorts of companies (see Figure 2). This becomes problematic when knowing an employee’s exact location does not relate to supervising their work or when the tracking continues also off the clock, a practice that has been controversial (U.S. Courts Opinions, 2015). In this sense, shifting the locus of surveillance from either the physical or the digital sphere to an integrated observation mode, as well as extending the desire for control to an employee’s private life and private property, creates additional tensions.

### Using surveillance information for subtle changes in the social dynamics at work

Some connected workplace surveillance solutions go far beyond registering an employee’s body functions or physical location. Companies—such as Humanyze (mentioned above)—have developed devices that use speech recognition and sentiment analysis that should enable employers to examine how and in what tones employees talk to one another, or how long and with whom they share their coffee or lunch breaks. Similarly, Walmart has patented a system named Listening to the Frontend (Jones et al., 2017), which monitors and filters specific noises (e.g., item scanners’ beeps or the rustling of bags), for recording and analyzing the conversations between employees and customers. The question of intentionality arises: What could the objectives of companies be to collect and scrutinize personal (e.g., sentiments, opinions, and tastes) and non-work-related information (e.g., an occasional chat at the coffee corner)? While we cannot (yet) know for certain, we posit that one intention could be to gather data that serve as a basis for designing and implementing subtle behavioral cues in the workplace.

Paternalistic approaches that help to trigger modifications of attitudes, perceptions, motivations, and actions are gaining traction in many different areas, among others, also at work (Feng et al., 2019; Pellegrini and Scandura, 2008). *Nudging*—understood as a concealed way of using design, information, and interaction elements to guide behaviors in online and offline environments (Ho and Lim, 2018; Johnson et al., 2012)—is often presented as a non-coercive way to adjust a person’s behaviors without necessarily affecting their choice options (Sunstein, 2014). Promising to be an alternative approach to overcome areas where traditional management practices based on hierarchy, legitimacy, and power (Montgomery, 1980) have been proven to be ineffective, nudging is not limited to monitoring and controlling a task’s completion but equally seeks to ensure that a task is continually done in the way desired by the employer. Accordingly, next-generation work surveillance systems will not be restricted to collecting information about performance, behavior, or personal characteristics (Ball, 2010), as was the case in visual surveillance or computerized surveillance, but extend their scope to modifying the social dynamics at work through behavioral strategies, such as nudging, gamification, and others (e.g., based on pressure, persuasion, or seduction). These new characteristics of work surveillance (see Table 3) have implications for control theory—as will now be discussed.

**Table 3.** Studies that exemplify the emphasis of moving beyond monitoring work to employees’ mindset and habits.

Issue	Data collected	Exemplary studies
Analyzing employees’ sentiments, opinions, and tastes	Voice, personal social media posts, email, other written communication, etc.	Alamsyah and Ginting (2018) Dai et al. (2013)
Applying subtle behavioral change strategies (nudging, gamification, etc.)	Responses to cues, activity patterns, communication patterns, etc.	De Moya and Pallud (2020); Fort et al. (2016); Hirsch (2019); Lord Ferguson et al. (2019)

## The conception of control in the connected workplace

In the Taylorist worldview, control has always been a key element. As noted, early work surveillance types centered on marking presence and the gaze-based control of laborers’ work outcomes and behaviors. Computerization helped to extend control to the digital world, for instance, by introducing digital performance dashboards. A fundamental assumption of this conception of control has been that work performance is most effectively managed and enacted through social contracts, agreements, and arrangements (Huang Chua and Myers, 2018; Kirsch et al., 2002). Following this perspective, *control* refers to actions taken by an employer to measure, evaluate, and alter employees’ work outcomes and behaviors, mainly through rewards and penalties (Eisenhardt, 1985). *Outcome controls* monitor the compliance of an employee’s *products of work* to predefined milestones, quality standards, or expected performance levels (Gallivan, 2001; Soh et al., 2011). *Behavior controls* seek to ensure that an employee’s *work process* aligns with the conduct and behaviors desired by the workplace (Kirsch, 1996). This is done, for instance, by construing an employer’s expectations through job descriptions, professional conduct policies, or a code of ethics (Gotterbarn et al., 1999), by mandating the use of project and process methodologies (Maruping et al., 2009), or by organizing meetings, conference calls, and walkthroughs (Choudhury and Sabherwal, 2003). Controls are not necessarily formal, that is, they do not necessarily rely on institutional power to effectively encourage a particular outcome or behavior (Ouchi, 1980). Several studies find formal controls to be problematic (Huang Chua and Myers, 2018; Lim et al., 2011) or more effective when combined with informal controls (Remus et al., 2020; Soh et al., 2011) that are enacted with minimal reliance on hierarchy; that take advantage of shared values, philosophy of work, and problem-solving approaches; or that regulate behaviors and outcomes based on group control or self-control (Keil et al., 2013; Tiwana and Keil, 2009). To examine how this more social conception of control affects behaviors and outcomes, most of the studies in this research stream have concentrated on visual surveillance practices and a very specific work environment: outsourcing or working in IS development projects (Choudhury and Sabherwal, 2003; Huang Chua and Myers, 2018; Kirsch, 1997; Maruping et al., 2009; Remus et al., 2020; Soh et al., 2011). Projects represent a special organization of work in the sense that the structures and collaborations are temporary, the work routines are less repetitive, and the desired product (and sometimes even the process) is typically well documented. It is common that the social dynamics and norms that are developed and applied in a project differ from those outside it (Lindgren and Packendorff, 2006).

**Figure 3.**
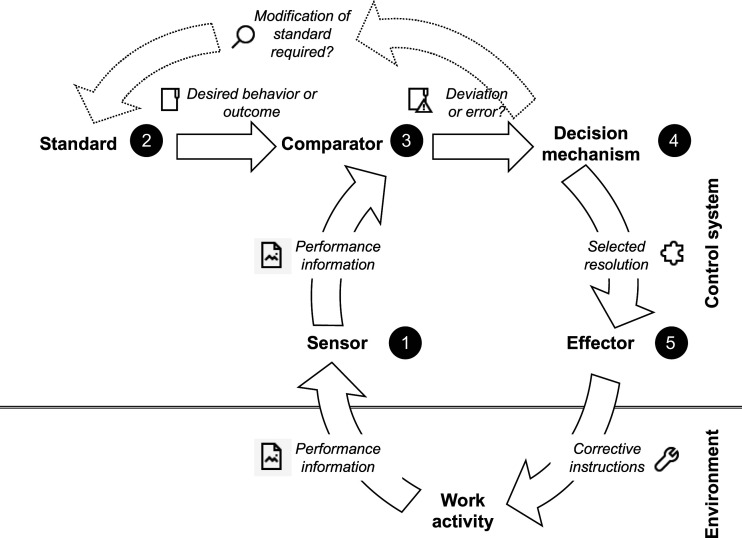
Components of a control system and their interrelationships (adapted from Lord and Hanges (1987)).

Seeking to understand and model—in more general terms—the governing principles of regulatory and purposive systems, a second research stream has taken a cybernetic view of control (Campion and Lord, 1982; Powers, 1978). Along the *organization-as-a-machine* analogy, here, context shrinks to a closed and well-defined set of mappings between inputs and outputs as well as causes and effects (Lyytinen, 2011). Similar to how a thermostat regulates the room temperature, Lord and Hanges (1987) posit that five distinct components are sufficient to control most everyday work settings: (1) a sensor that measures or gathers performance information, (2) a standard or goal that the employer seeks to maintain or achieve, (3) a comparator that contrasts the sensed information to the standard, (4) a decision mechanism by which certain actions are proposed for reducing any discrepancy between the sensed information and the standard, and (5) a response mechanism or effector that implements these actions and interacts with the environment. As illustrated in Figure 3, control is perceived as a feedback loop that starts when a sensor registers performance information from the environment and feeds it to the comparator, which compares this information to the standard. Suggesting different problem-solving or resolution strategies, the decision mechanism is set in motion in the case of a discrepancy, either instructing the effector to implement and/or communicate the selected resolution strategy to the work environment, or to adapt the standard and goals if they were unrealistic, misaligned, or erroneous. Providing a dynamic perspective on the interdependence of goal-setting and performance measurement, a control system in this sense continually senses and compares inputs to desired outputs and initiates further actions when it identifies discrepancies (Sandelands et al., 1991).

Such a mechanistic interpretation of control has faced fierce and sustained criticism for being too centered on controllable and measurable stimuli and ignoring the importance of and guiding role of social contracts. Sandelands et al. (1991) also comment on the fact that feedback can be given even when no discrepancy exists (e.g., for encouragement or commendation). Although we agree with the criticism that this worldview is reductionist and unable to reproduce the full complexity of human social interaction (Nach, 2015), we deem it extraordinary useful for analyzing the changes in work surveillance regimes. Based on the cybernetic view of control, Table 4 summarizes how control is implemented in the different work surveillance modes; this is our starting point for reflecting on their possible social consequences, which we will now discuss.

**Table 4.** Implementations of control in different work surveillance modes.

	Visual surveillance	Computerized surveillance	Connected surveillance
*Environment*	The locus of surveillance is mostly offline, in the premises of the workplace	The locus of surveillance is mostly online, on the infrastructure provided by the employer	A combination of offline and online tracking during work and leisure, and sometimes on the employee’s private infrastructure
*Sensor*	A human overseer (e.g., shift supervisor, office manager, project manager, chief nurse, and prison guard), or group control or self-control	Computer program (e.g., data tracking through event logs, access history, and screen time)	Embedded and intelligent systems (e.g., sensor networks, wearable devices, body implants, and data tracking)
*Standard*	Formal or informal work-related outcome controls: Predefined milestones, quality standards, or expected performance levels	Mostly formal work-related outcome and behavior controls similar to the previous surveillance mode	Formal work-related outcome and behavior controls combined with standards for individual characteristics (e.g., personal fitness, stress, and psychological well-being) or non-work-related factors (e.g., personal sentiments, opinions, and tastes)
	Formal or informal work-related behavior controls: job descriptions, professional conduct policies, or a code of ethics; project and process methodologies and walkthroughs		
*Comparator*	A human overseer supported by technical measures (e.g., punch cards and alarm clocks)	A human overseer supported by (a) computer program(s) (e.g., CPMS, data mining, and analytics solutions)	Machine learning algorithms that compare sensed information to predefined standards and thresholds
*Decision mechanism*	Human judgment	Computer-supported decision-making; the final decision is taken by a human	Algorithmic decision-making, that is, decisions are taken by a computer based on learned patterns
*Effector*	A human overseer communicates corrective instructions, or group regulation or self-regulation	A human overseer communicates corrective instructions, or group regulation or self-regulation	Corrective instructions are communicated to embedded systems, which either take direct action (e.g., informing employees) or indirectly nudge them

## Social consequences and impacts on work performance

The shift from human oversight and judgment to algorithmic decision-making and the systematic accumulation of employees’ individual characteristics (e.g., personal fitness, stress, and psychological well-being) or non-work-related information (e.g., personal sentiments, opinions, and tastes) in connected workplaces will inevitably lead to new consequences. To date, there is little firm evidence on how the described changes in work surveillance will play out. Possible implications are often discussed from a business perspective, such as the role of managers in light of their gradual replacement by algorithms (Bader and Kaiser, 2019; Lindebaum et al., 2020), or the accountability, transparency, and discrimination issues faced by organizations that implement algorithmic decision-making (Ågerfalk, 2020; Newell and Marabelli, 2015; Watson and Nations, 2019; Young et al., 2021). We now ask: How do increased and new work surveillance types impact on ordinary employees? This question has received very little attention (Giermindl et al., 2022). Although we still lack solid data to explain what connected workplaces will imply for the future of work, normative and speculative research can be useful for developing a forward-looking research agenda (Baptista et al., 2020).

A key element of connected surveillance is its replacement of control measures performed by humans with devices and algorithms that appraise work activities, inform employees, and execute certain predefined resolution strategies. As Clarke (2019, p. 60) notes, “*genuine relationships between organizations and people are replaced by decision-making based on data that has been consolidated into digital personae.*” According to Lyytinen (2011), such a fully rationalized and formalized management ideology may create the illusion of having more control and may justify the unabated expansion of data collection. Thus, algorithmic decision-making, as a part of implementing control in the connected workplace, has become a subject of heated scholarly debate (Marabelli et al., 2021; Marjanovic et al., 2022). On the upside, algorithmic decision-making holds the promise of being more efficient, scalable, and consistent than humans in responding to work deviations and errors (Wisskirchen et al., 2017); on the downside, it carries the risk of hidden normative decisions (Marjanovic et al., 2021a) because the data used for clustering, training, and testing algorithms may contain distortions that are seemingly objectified by the process itself (Benbya et al., 2021; Marjanovic et al., 2018).

If the causalities behind the correlations in algorithmic decision-making are not verified, there is a strong risk of unintentional systematic discrimination, which inevitably impacts on job satisfaction and well-being at work (Bhargava et al., 2021), especially if employees who feel discriminated against do not have appropriate ways to reconstruct or appeal against an automated decision (Wagner, 2019). Whether employees under current privacy laws—such as the aforementioned GDPR—have a *right to an explanation* on the grounds on which and how such an automatized decision mechanism works remains a matter of judicial dispute (Wachter et al., 2017). The research has shown that procedural fairness (i.e., an employee’s perception of being treated (un)fairly by their employer) is crucial to building a trusting employer–employee relationship in the workplace (Carpenter et al., 2018). Not having access to the code that evaluates the quality of one’s work or that decides one’s promotion seriously damages this trust relationship (Bankins et al., 2022), which is why algorithmic decision-making is often perceived as being “*demeaning and dehumanizing*” (Lee, 2018: p. 13).

Yet even if access to these algorithms is granted, will ordinary employees be able to understand the code or the instructions? Ananny and Crawford (2016) note that seeing a code is not the same as knowing how it works. Thus, trust-building is not achieved by simply granting access to a code; as Dolata et al. (2022) posit, algorithmic decision-making requires considerations that go beyond purely technical measures. Yet this stands in stark contrast to policy and industry efforts that place their hope in technical responses—such as privacy-by-design (Nussbaumer et al., 2022)—without interrogating the expansionary practice of sensing and standardizing the outcomes, behaviors, and personal characteristics needed to make the connected workplace surveillance a reality (Kellogg et al., 2019). For instance, at the Amazon Fulfillment Center in New York, to take a toilet break, warehouse workers must log a “time off task” (Jabsky and Obernauer, 2019). Thus, we presume that connected workplaces will expand the level of formal control and gradually replace informal social control where possible. As control over employees' performance, behaviors, and sentiments continues to expand, even the smallest aspects will need to be formalized in the future so that connected workplace solutions can function properly. This brings us to our first testable proposition:

*Testable proposition 1: A connected workplace leads to over-formalized control.*

While sensor-based and AI-based surveillance tools are not yet as adaptable and empathetic as human overseers may be (Mettler and Wulf, 2019), one distinct, irreducible characteristic of a connected workplace—from an employer perspective—is that surveillance of work activities and work environments can be permanent and omnipresent (De Moya and Pallud, 2020). Building on the assumption that surveillance at work is—first—a necessity and—second—a taken-for-granted part of working life (Ball, 2010), employers are often not aware of excessive monitoring’s negative consequences or dismiss them, considering monitoring to be a good management practice. According to Burke (2004), the wish to measure and appraise performance has taken on a cult-like status, which has not only replaced purpose in modern organizations but also uses coercive persuasion and indoctrination to vindicate actions and claims.

While setting objectives, reviewing performance, and gathering information on the quality of their work is something that employees should expect and to a certain extent accept, as Anderson et al. (2017) argue, employers walk a fine line between two extremes: the need to share information and the need to protect information. Tensions generally arise when a mismatch occurs. This is the case when surveillance goes beyond what is reasonable or necessary (Ball, 2010), or when it compromises working practices, negatively affecting autonomy and personal integrity (Pedersen, 2020). For most employees, it often remains inapprehensible why their employer needs their personal and non-work-related data for the purpose of performance appraisals and managerial oversight (Park et al., 2021). Even if an employer would disclose the reason(s) why a specific surveillance technology is adopted or why certain information is collected, ethical issues remain regarding privacy, accuracy, property, and the accessibility of the gathered information (Mason, 1986). The issues caused by connected workplace surveillance for privacy alone are extensive (Bhave et al., 2020)—it not only touches on information privacy but also extends to questions concerning privacy and the human body, privacy in social relationships, and/or privacy and personal space. As Bloustein (1964) notes, privacy is a matter of dignity.

In the context of connected workplaces, dignity implies that all individuals, whether they are employers or employees, should consistently receive respectful treatment and never be regarded as mere tools or objects. Hence, it places a special obligation on employers to offer meaningful and respectful work conditions (Bowie, 2019) and to apply responsible digitalization (Leidner and Tona, 2021). Several concepts—such as equal and fair treatment, autonomy, or freedom of expression—are connected to work dignity (Tiwari and Sharma, 2019). Yet several studies have demonstrated that less sophisticated CPMS may already undermine an employee’s dignity (Alder, 1998; Snyder, 2010; Westin, 1992). Studies also show that constant and abusive surveillance of employees creates a toxic work climate (Men et al., 2022), which has been particularly noticeable in precarious employment types such as those of gig workers, call center agents, and warehouse packers (Bain and Taylor, 2000; Ball and Margulis, 2011). Thus, we presume that excessive control in connected workplaces significantly affects an employee’s dignity and sense of being respected as a human being, which leads to our second testable proposition. This leads to our second testable proposition:

*Testable proposition 2: Expanding formalized control leads to a loss of dignity in the workplace.*

While connected workplaces potentially lower the trust of and respect toward workers, paradoxically, there is significant evidence that a trustful work relationship is necessary for formalized and automated management approaches to work effectively (Kulik and Ambrose, 1993; Scott, 1980; Sia et al., 2002). Several studies have shown that dehumanized and undignified work environments grounded in permanent and omnipresent surveillance can cause serious harm and can lead to anxiety, stress, and depression among workers (Ball and Margulis, 2011; Carayon, 1994; George, 1996; Tarafdar et al., 2019). A recent example that has received media attention is the practice of algorithmically setting conveyor belt speed based on biometric data, pushing employees to the limits of overwork (Ongweso, 2022). Negative effects are further exacerbated when, in the event of performance shortfalls or alleged misconduct, algorithms initiate punishing or sanctioning interventions, rather than providing constructive and developmental feedback.

According to Anteby and Chan (2018), the managerial efforts to expand control ultimately give way to a self-fulfilling cycle of coercive surveillance: in response to increased surveillance, employees often develop evasive tactics at work, which again justifies the expansion of managerial oversight. Thus, employees have few options to fight back. As Scott (1985, p. 29) notes, resistance can take many forms, including “foot dragging, dissimulation, false compliance, pilfering, feigned ignorance, slander, arson, sabotage and so forth.” Knowing that one is being monitored and appraised by a machine every second of the workday can lead to the counter-productive effect of actively resisting, evading, or tricking the system. For instance, Marx (2003) describes different strategies for neutralizing or subverting an employer’s excessive collection of personal information, such as avoidance, piggybacking, or distorting moves. Ferneley and Sobreperez (2006) showed that growing dissatisfaction and resentment among workers result in different workaround types. More recently, Mettler and Wulf (2020) found that reputational and monetary rewards underlying data-driven corporate wellness programs encourage social cheating and may therefore jeopardize the de facto undertaking of stimulating healthy behaviors.

Since connected surveillance erodes self-determination, autonomy, and choice, employees will need to spend some time and be more creative if they are to identify potential gaps and workarounds. More skills and effort will be needed to evade connected surveillance at work (Marchant, 2019). Thus, we presume that the expansion of control will have a counter-intuitive effect and will potentially lower a firm’s overall productivity owing to anxiety and stress, or owing to evasive tactics to circumvent excessive surveillance practices. This brings us to our final testable proposition:

*Testable proposition 3: Defensive reactions to connected surveillance lead to performance loss in the workplace.*

## A proposition for a research agenda

Having set out our testable propositions, we will now propose an employee-centric research agenda for the connected workplace (see Table 5). We will then identify key questions that future research should investigate relating to each testable proposition and will clarify the link to the control theory, as described. We will also suggest directions of inquiry for researchers to engage with these questions, based on a critical, behavioral, or design-oriented research perspective.

**Table 5.** Propositions for an employee-centric research agenda on the social consequences of connected surveillance.

Testable propositions | possible manifestations	Main links to control theory	Research questions	Possible directions of inquiry from a critical, behavioral, or design-oriented lens
The proposition regarding the over-formalization of control	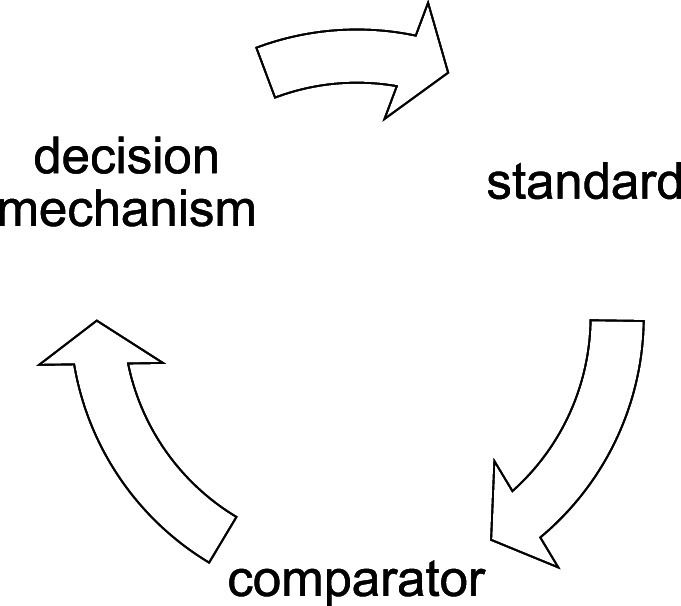	⁃ What are common moral and ethical conflicts when algorithms appraise humans?	⁃ Critical: The acceptability of algorithmic standards for evaluating humans; ethical dilemmas from a double standard for overseeing and ordinary employees
⁃ The feeling of not being treated as a human being (Lee, 2018)		⁃ How does the introduction of connected workplaces affect employee–employer relationships?	⁃ Behavioral: Effects of closed-loop algorithmic control on employee–employer trust relationships; the relationships among choice (mandated use, quasi-mandated use, and free choice), co-determination, and transparency on the acceptance of algorithmic management
⁃ The feeling of having no influence on decisions (Wagner, 2019)		⁃ How can we design systems that are fair, empathetic, or even anthropomorphic in their appraisal of employees?	⁃ Design: The development of effective governance mechanisms for algorithmic decision-making; empathetic and anthropomorphic design of algorithms
⁃ The feeling of not being treated fairly (Carpenter et al., 2018)			
⁃ A loss of trust in the employer (Bankins et al., 2022)			
The proposition regarding the loss of dignity in the workplace	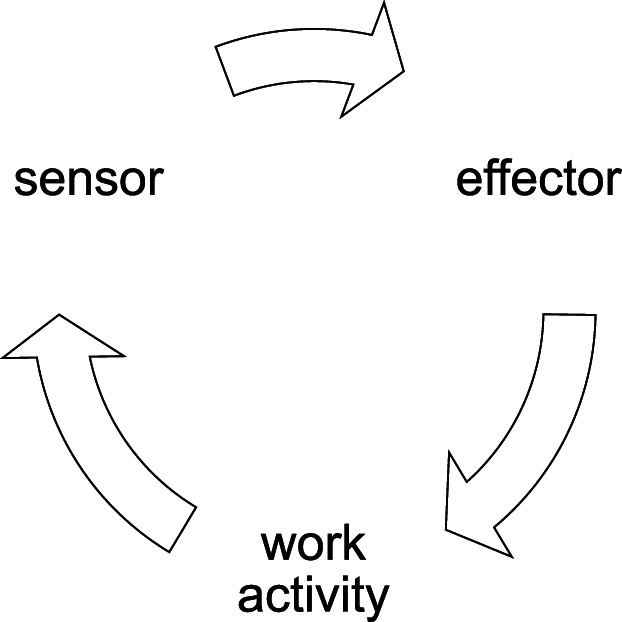	⁃ To what extent do employees have to endure manipulative, humiliating, or coercive surveillance practices in their workplace?	⁃ Critical: Exposing common practices in connected workplaces that involve manipulation, humiliation, or coercion of employees; different algorithmic bias types
⁃ A perceived loss of privacy, autonomy, and personal integrity (Leidner and Tona, 2021)		⁃ What positive and negative effects do connected workplaces have on employees’ personal situations?	⁃ Behavioral: Effects on employees’ health and well-being caused by excessive work surveillance; impacts of varying degrees of transparency, autonomy, and privacy of algorithmic management on employees’ dignity and the overall work climate
⁃ The perception of working in a toxic work environment (Men et al., 2022)		⁃ How can we design systems that are more context-sensitive and adaptable in their responses to employees’ personal situations?	⁃ Design: Minimal invasive and employee-centric data collection techniques; design of contextualized and adaptable algorithmic responses
⁃ The prevalence of depression, stress, anxiety, and other disorders (Tarafdar et al., 2019)			
The proposition regarding performance loss	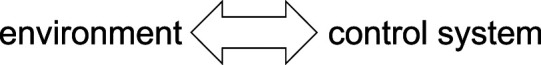	⁃ To what extent is connected surveillance acceptable in today’s working environment?	⁃ Critical: Developing arguments against the common perception that surveillance is justified and acceptable; ethical dilemmas that emerge from paternalistic approaches (e.g., gamification and nudging) for boosting performance and information disclosure
⁃ A dissociation from work and the development of resistant behaviors (Anteby and Chan, 2018)		⁃ How does the introduction of connected workplaces affect job performance?	⁃ Behavioral: Long-term impacts of connected surveillance on employees’ work performance; the relationship between the extent of abusive surveillance and the emergence of workarounds in connected workplaces
⁃ Looking for ways to outsmart the system (Mettler and Wulf, 2020)		⁃ What factors increase the likelihood of resistant and evasive behaviors among employees?	⁃ Design: Approaches for increasing happiness, not just data disclosure or system use
⁃ The active evasion of the work environment and of corporate control (Marchant, 2019)		⁃ How can we design systems that lead to a happier yet effective workforce?	

### Future directions relating to the proposition regarding the over-formalization of control

As noted, the feeling of being in a dehumanized workplace results from the fact that decision-making authority is gradually being transferred to machines, so that employees often do not understand how management decisions are taken (Ananny and Crawford, 2016) and what to do when they perceive decisions to be wrong (Bankins et al., 2022). A close look at the interplays among *standards*, *comparators*, and *decision mechanisms* in cybernetic control theory allows for the definition of various exciting research questions on moral and ethical conflicts of algorithmic justice (Marjanovic et al., 2022), fairness (Carpenter et al., 2018), and bias (Gupta et al., 2022) in the workplace. For instance, from a critical perspective, researchers could ask questions about the general acceptability of algorithmic standards for appraising humans and could discuss the implications of unequal treatment, particularly when companies adopt one standard for managers and one for ordinary employees (i.e., a double standard) (Greenwald, 2019). Better understanding closed-loop algorithmic control’s effects on employee–employer trust relationships (Lee, 2018) and how choice (or the lack of it), co-determination, and different degrees of transparency affect the acceptance of algorithmic management (Jarrahi et al., 2021; Marabelli et al., 2021; Watson and Nations, 2019) could be an avenue for behavioral researchers. From a design-oriented perspective, researchers could experiment with distinct approaches to the effective governance of algorithmic decision-making, for instance, whether today’s work environments would not be more productive with human-in-the-loop work configurations than with fully automated decision tools (Grønsund and Aanestad, 2020). Further, researchers could explore how to design more empathetic and anthropomorphic decision-making algorithms (Benlian et al., 2020).

### Future directions relating to the proposition regarding the loss of dignity at work

Since the IS community has mostly been concerned with the organizational and managerial ramifications of datification, sensorization, and AI, a myriad of research questions regarding employee dignity remains unanswered (Leidner and Tona, 2021). Taking cybernetic control theory as a starting point, such issues often arise from the interplays among a *sensor*, an *effector*, and a *work activity*, as well as around the questions what employee data are collected and how they are used (to benefit an employee, or to their detriment) to change attitudes, perceptions, motivations, and actions (Díaz Andrade and Techatassanasoontorn, 2021). The aforementioned case of Amazon exemplifies that contemporary work surveillance systems pay little attention to the dignity of ordinary employees, overstep personal boundaries, and sometimes even legal boundaries (U.S. Courts Opinions, 2015); thus, they often cause a toxic work climate (Men et al., 2022). Yet there is relatively little firm evidence that showcases abusive, manipulative, humiliating, or coercive surveillance practices in today’s workplaces. From a critical research perspective, uncovering and exposing undignified working conditions that stem from excessive data collection and subsequent algorithmic responses would be very beneficial to the research community and to society. Following the research into technostress (Ayyagari et al., 2011; Ragu-Nathan et al., 2008; Tarafdar et al., 2019), behavioral researchers could further shed light on how excessive work surveillance affects employees’ health and well-being, or on how more privacy, autonomy, and a more respectful treatment of ordinary employees could contribute to a better work climate. According to Gupta et al. (2021), algorithmic management approaches need to become more task-aware, that is, they need to formulate different responses to different situations or problems. Thus, developing more contextualized and adaptable algorithmic responses (Wang et al., 2020) that prioritize human dignity over corporate profits could be a point of entry for design-oriented research to counter the identified challenges. Further, we need new design approaches that not only respect and preserve privacy (Oetzel and Spiekermann, 2014) but also that are minimally invasive or that achieve the same outcome without data collection. In this context, a system’s utility—often the principal criterion for assessing design-oriented research’s quality (Winter, 2008)—could be measured by how sparingly data are handled and not just by how securely a system is designed to prevent unauthorized access or the repurposing of data.

### Future directions relating to the proposition regarding performance loss

Besides considering the interplays between different components of control theory, it is also crucial to not lose sight of the big picture: To what extent is a sensor-based and AI-based control system necessary? And: How does it affect individuals, groups, and organizations? Critical researchers could counter the commonly accepted narrative that work surveillance is irreplaceable for the productive operation of businesses (Ball, 2010). They could further highlight the moral and ethical issues of paternalistic approaches (such as gamification or nudging) regarding enhancing employees’ performance and information disclosure (Gal et al., 2020; Möhlmann et al., 2021; O'Donnell, 2014). An interesting avenue for behavioral researchers could be to examine the long-term effects of connected surveillance on employees’ work performance and which factors or configurations facilitate the emergence of workarounds and evasive behaviors (Mettler and Wulf, 2020). As discussed, a lot points to a counter-intuitive effect that increased and more sophisticated surveillance practices lower a firm’s productivity. For design researchers, this raises new questions about how to fundamentally design systems that not only get employees to share personal data or accomplish certain tasks but also that genuinely improve their well-being and job satisfaction, so that they do not need to be pushed or nudged to be more productive.

## Conclusion

The pattern we have described as *connected surveillance* comprises much more than just spying on and controlling employees. Fundamental rights and human dignity are threatened by the implementation of ever-more-comprehensive types of digital employee monitoring, and the question arises what researchers can do to enable understanding of the negative consequences and to prevent damaging effects on salaried employees and on society. We looked at how datification, sensorization, and AI are changing the ways in which companies control their workforce and what possible social consequences these have for ordinary employees. Taking an employee-centric perspective, we proposed a research agenda for critical, behavioral, and design-oriented scholars who wish to further explore the identified issues.

A limitation of this article is the lack of a detailed consideration of how existing legal frameworks and the enforcement of laws counteract abusive surveillance and inhumane work conditions (Short and Toffel, 2010). We have deliberately only hinted in certain passages that legal frameworks—such as the GDPR—tend to favor companies yet have ignored the fact that, in some countries, labor law is heavily weighted toward workers’ rights. Nonetheless, the regulation of new technologies poses massive challenges to many legislators, who therefore tend to privilege industry self-regulation (Gal-Or et al., 2018; Terlaak, 2007). In the absence of explicit sanctions and a willingness to pursue misconduct, effective self-regulation seems unlikely (Bowen, 2019) and continued arbitrary decisions by companies as *private governments* seem most likely (Anderson, 2017).

The introduction of connected workplace surveillance will continue, leading to ethical, social, and/or economic contradictions and ambiguities. With our research agenda, we have identified issues that require special attention and the skill set of researchers with a socio-technical orientation. Who other than IS scholars possess the essential expertise to comprehend both the intricate technical aspects and social implications of connected workplaces? Therefore, we ask the IS community to further uncover contradictions, tensions, untruths, or delusions about connected surveillance and closed-loop algorithmic control as well as to gather empirical evidence that the narratives used by tech companies and employers do not always correspond with reality or keep the promises they make. We urge not to uncritically repeat such narratives, since the connected workplace is more likely to serve the interests of the powerful. In the spirit of research like the Scandinavian “trade-unionist approach” (Bødker et al., 1987; Hedberg, 1980; Iivari and Lyytinen, 1998; Sandberg, 1985), which might have been somewhat forgotten over the years, we propose considering how the power politics of permanent and omnipresent surveillance affect the working conditions and well-being of ordinary employees, particularly those who due to certain life circumstances cannot easily choose or switch employers. In light of the flexibilization of work and the emergence of digital nomadism as the antithesis of geographically and time-bound labor (Wang et al., 2020), we should now fundamentally challenge the ways in which work relationships are being portrayed, as well as the exact purposes of knowing, controlling, and modifying work behaviors.
